# Intestinal HAdV Infection: Tissue Specificity, Persistence, and Implications for Antiviral Therapy

**DOI:** 10.3390/v11090804

**Published:** 2019-08-30

**Authors:** Karin Kosulin

**Affiliations:** Molecular Microbiology, Children’s Cancer Research Institute, Zimmermannplatz 10, 1090 Vienna, Austria; karin.kosulin@ccri.at; Tel.: +43-1-40470-4875; Fax: +43-1-40077-64890

**Keywords:** human adenovirus, intestinal infection, tissue tropism, recombination, persistence, antiviral mechanisms

## Abstract

Human adenovirus (HAdV) causes infections predominantly in early childhood and the tissue tropism of specific HAdV species determines the clinical manifestation, including infections of the gastrointestinal tract, respiratory tract, and keratoconjunctivitis. Why HAdV shows such a tropism has not yet been fully elucidated, but in the intestine different mechanisms for virus entry or resistence to immune modulatory factors have been described. Recently identified antiviral strategies by interferons provide evidence about the repression of *E1A* and maybe even promote HAdV persistence. The presence of HAdV in a persistent status in the gut is of importance in the setting of pediatric stem cell transplant recipients where HAdV detection in stool usually preceds clinical signs and severe infections are related to mortality. The reactivation of persistent intestinal HAdV infections in these patients needs further investigation also with regard to successful therapy options. In addition, several newly identified recombinant HAdV types have been isolated from stool samples, thus raising the question of possible recombination events in the gut. In this review, intestinal HAdV infections are discussed in relation to the tissue tropism, persistence, recombination, and new in-vitro models to enhance the knowledge about virus–host interactions and support the development of new treatment approaches.

## 1. Introduction

The best-documented sites of human adenovirus (HAdV) replication and infectious virus production are the intestine, the lower airway tract, and the eyes where the virus can cause a variety of clinical manifestations ranging from mild to severe diseases [1]. Epithelial cells are known to provide permissive host cells for effective HAdV replication [1,2,3] and, in individuals with an intact immune system, HAdV infections are usually cleared by virus specific T cells [4,5]. Following primary infection, most commonly in early childhood, HAdVs can persist in a latent state [1,2,6]. Persisting HAdV DNA was first described in tonsillar and adenoidal tissue [7]. Later, detailed analyses identified the virus in the mucosal T lymphocytes derived from children [6,8]. Moreover, there is strong evidence for HAdV persistence in the gut since shedding of the virus into the stool from asymptomatic individuals was described [1,9] and HAdV was found in T cells isolated from the intestinal lamnia propria [10]. Although the mechanisms for HAdV persistence and reactivation are unknown, antiviral mechanisms mediated by the innate immune system [11,12] might play a role in the maintenance of virus persistence [13]. An interferon mediated repression of virus progeny production is described for epithelial cells [14]. This could also be a possible regulation for HAdV persistence in T lymphocytes but, so far, there is no evidence for this. In patients lacking a functional immune system—e.g., in pediatric allogeneic human stem cell transplant (HSCT) recipients—HAdV replication begins mainly in the intestine where a reactivation of viral persistence is assumed [2,15,16,17,18,19,20,21]. Severe disease can be a result of intestinal HAdV reactivation in the immunosuppressed host and a successful antiviral therapy is still missing. Thus, the intestine, representing a significant part of our immune system, might provide an important site to study virus–host interactions. In-vitro systems with primary intestinal cells could provide suitable models to understand the mechanism for HAdV persistence and reactivation, but also for drug screening of potential new antivirals.

HAdVs represent a large family of genetically diverse pathogens and are divided into seven species (A–G) currently comprising 103 types [22], listed in Table 1. During the last decade, a vast number of recombinant types have been identified due to improved sequencing technologies elucidating the whole viral genome. However, despite the number of HAdV types has increased rapidly, data about the clinical impact of recombinant types are still rare [23,24,25,26]. The individual HAdV species and types show a broad tissue tropism and also different receptors for virus entry into the host cell are being described. Infections in the gastrointestinal tract are mainly caused by species F, in the respiratory tract by species B, C, and E, and conjunctivitis is caused by species B, D, and E. Interestingly, only the species F types cause intestinal diseases in children [1,27], whereas under immunosuppression also other types are associated with the occurrence of severe gastroenteritis [15,17,20,28]. The underlying mechanism of this species-specific phenomenon and whether a distinct immune regulation plays a role in this context needs to be investigated.

## 2. Species-Specific Intestinal HAdV Infections—A Matter of Virus Entry or Immune Modulation?

The highly variable tissue tropism for HAdV may be attributable to the different receptors facilitating virus entry. Moreover, regulation by distinct antiviral strategies and the host’s immune response might also be possible and cannot be excluded. Most of the HAdV species (A, C–F) enter the host cells via recognition of the fiber protein on the viral capsid by the coxsackie-adenovirus receptor (CAR) [29]. CAR is widely expressed on epithelial cells [30], whereas many tumor cells lack the virus specific receptor [31,32]. Species B types are recognized by the more ubiquitously expressed surface marker CD46, first described in 2003 [33]. The two enteric types HAdV-F40 and HAdV-F41 use CAR for virus uptake, but differences due to a short fiber protein, present only on the virus surface of species F types, are suggested. Resistance to inactivation in acidic conditions of the short fiber protein might be the cause for the enteric tropism and chimeric HAdV-C5 with the HAdV-F40 fiber show a better uptake into intestinal epithelial cells than wildtype HAdV-C5 [34,35].

**Figure 1:**
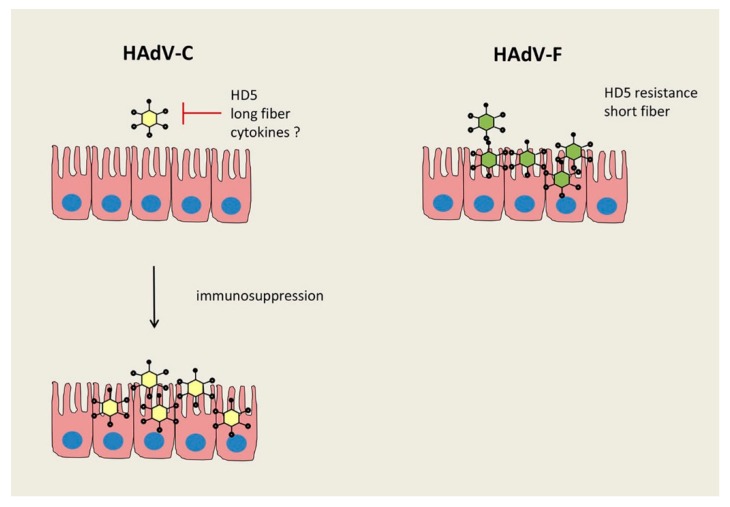
Different regulation of intestinal HAdV infections by species C and F. The production of HAdV-C types is regulated by human defensin 5 (HD5), whereas HAdV-F types are resistant to HD5, secreted by intestinal Paneth cells. A higher stability of the short fiber protein on HAdV-F types is suggested to also play a role for the frequent gastrointestinal tract infection by these species. Another important regulation might be due to specific cytokines (e.g., IFNα, β, γ, or λ) and their antiviral capacity.

Whether the different fibers contribute to the well described prevalence of acute gastroenteritis caused by species F types [1,36], or other species dependent defense mechanisms in the human gut, remains unclear. Paneth cells in the small intestinal epithelium produce innate host defense peptides, the α-defensins, including human defensin 5 (HD5) and human defensin 6 (HD6), to limit invasive attacks by bacteria and viruses [37,38]. Recently, an enhanced shedding of mouse adenovirus 2 (MAdV-2) induced by α-defensins was reported and diverse anti-viral capacity by defensins have been described among different MAdV types [39]. Furthermore, HAdV types of species A, B, and C are neutralized by HD5, whereas infection with species D and F types seem to be resistant to neutralization by the chemokine, as tested in a lung epithelial cell line and in primary intestinal cells [40,41]. These data show variations between the different AdV types, both in mouse and human cells, but the detailed mechanism of this phenomenon is not known. However, an adaptation of specific viruses to these host proteins to utilize them for promoting their own replication has been suggested [41,42]. Moreover, it might be of interest to understand the commonly occurring gastroenteritis as being induced by the species F types [36], whereas a predominance of persistent species C types was detected in intestinal tissue of immunocompetent pediatric patients with rare cases of F types [2]. Only under immunosuppression HAdV species C results in vast virus progeny production in the intestinal epithelium, causing severe gastroenteritis with high virus loads in stool [15,20,21,28,43,44]. These findings corroborate the intestinal epithelium as a general site for the replication of various HAdV species though the HAdV replication might be regulated by host defense mechanisms in a strong species dependent manner (see Figure 1). It is feasible that several host proteins can affect this tissue tropism and resistance to α-defensins seems to be one of them. Whether other factors also play a role in controlling virus infection in this context, like specific interferons, preferentially expressed in epithelial cells [45,46], has not been investigated.

## 3. Intestinal HAdV Persistence and the Relevance of Antiviral Strategies Mediated by Interferons

HAdV persistence was originally described in adenoids and tonsils [7], whereas the persistence of this virus in the gut has been suggested for decades. Already in the 1980s, the shedding of HAdV into the stool several months after any clinical signs of the infections are gone was reported [1,6,9,47]. Later, frequent findings of HAdV in stool specimens derived from immunosuppressed patients with diarrhea and HAdV associated mortality, including pediatric HSCT-recipients [20,21,28] and HIV patients [48,49], were reported. Thus, the intestinal tract might be a site of HAdV reactivation under immunosuppression. Severe invasive infections can be a consequence of the reactivated virus when a functional immune defense is lacking. Moreover, HAdV persistence in pediatric HSCT-recipients, already prior to transplantation and immunosuppression, was shown to correlate with a high risk for the later onset of viremia and invasive infections [2,19]. This supports the notion that HAdV persistence in the intestine may serve as a predictive marker for high-risk patients post-transplant.

A screening of biopsies derived from immunocompetent pediatric patients (*n* = 143) undergoing elective upper and lower endoscopy of the gastrointestinal tract for a variety of indications revealed one-third HAdV positive patients with the highest prevalence of HAdV in the ileum [2]. In the colon and the jejunum, the virus was also found at high instances; whereas in the stomach, duodenum, and rectum lower frequencies were observed. Overall HAdVs detected in the gastrointestinal tract the species C types were most prevalent, with low virus copy numbers of 10E3 per million cells [2]. Another study reported also high frequencies of HAdV in the ileum but with a predominance of HAdV-E4 [10]. Interestingly, in both studies, HAdV persistence was not found in the epithelium but in the lamnia propria, consisting of intestinal lymphocytes. Due to these observations one might assume a preferred route of infection via the basolateral side in the gut. Another intriguing question is how HAdV enters the body and is able to reach the gastrointestinal tract. It is well known that not only the typical enteric HAdV species F, but all other species, can infect intestinal cells. Moreover, the frequency of HAdV persistence in 30% immunocompetent children seems to decrease with age and a mother-to-child transmission could be feasible. Apart from these findings in intestinal biopsies, the knowledge about HAdV persistence in the gut is limited to clinical data from stool analyses but in-vitro analyses are still missing.

How interferon levels may influence HAdV persistence was reported by Zheng et al., exploiting an in-vitro infection model [14]. HAdV replication can be influenced by interferon (IFN) α and γ targeting several adenoviral early genes like *E1A*, *E1B*, or *E4ORF3*, whereas in tumor cell lines these effects seem to be abrogated [14,50,51]. In human fibroblasts and primary human lung epithelial cells IFN-α and IFN-γ efficiently reduce virus replication by repression of *E1A* expression. Interferons promote the binding of E2F/Rb family proteins in the *E1A* enhancer region blocking the recruitment of the cellular GA-binding protein (GABP) transcription factor, which is necessary to initiate *E1A* expression. This mechanism was observed in cells with an established persistent HAdV infection and the blocked virus replication could be reverted by withdrawal of IFN-γ [14]. However, the specific effector molecules triggering the binding of E2F/Rb family proteins to the *E1A* enhancer region are still unknown. Interestingly, E1B-55K has the capacity to block interferon inducible genes to facilitate viral replication [50], thus providing a viral defense mechanism. These data provide evidence of a strong influence of interferons on the regulation of HAdV replication. The balance of how the cytokines inhibit virus production while HAdV shows properties to evade the immune system needs further investigation. In addition, it has to be considered that type I and II interferons are mainly secreted by lymphocytes under stimulation with pathogens, whereas epithelial cells usually produce type III interferons [46].

Another model for HAdV persistence exploited tumor cell lines derived from T lymphocytes and established a long lasting HAdV replication at low levels in Jurkat and PM1 cells [52]. In addition, the presence of low amounts of virus progeny production over several months provides evidence for a carrier state infection in lymphocytes in this model. In studies reporting HAdV persistence in patient samples, the virus was always found in lymphocytes [6,8,10,53]. Their constitutive interferon secretion might be a prerequisite for the establishment of virus persistence through an initial suppression of virus replication. Moreover, it is intriguing that the inhibitory effect of immunosuppressive agents on specific cytokine expression triggers HAdV reactivation in the gut of HSCT-recipients. Persistence in intestinal lymphocytes was observed [10], but efficient HAdV replication in these cells has not been reported so far. Clinically used repressors of interferon activity are glucocorticoids or calcineurin inhibitors targeting the expression of several cytokines (e.g., IFN-γ, IL-2, IL-4, and TNF-α) [54,55]. Radke et al. mentioned the possible influence of immunosuppressive agents to support HAdV production in lymphocytes of HSCT-recipients [13]. Furthermore, one might speculate that the enhanced infection of the gut epithelium under immunosuppression, as it was found in pediatric HSCT-recipients [2], could be the result of increased virus replication and progeny production in intestinal lymphocytes by repressed cytokine secretion.

In a humanized mouse model harboring human leukocytes, 2-month post HAdV infection asymptomatic infection was observed. In this in-vivo model only early viral gene expression of E1A was present in blood and bone marrow but absent in the liver, spleen, thymus, and lymph nodes, whereas late viral gene expression of hexon was not expressed at any site [56]. Maybe the inhibition of viral gene expression by interferons is more pronounced in tissues compared with blood and bone marrow due to an enhanced antigen presentation at local infections. In the study of Roy et al., intestinal lymphocytes were tested for viral DNA but also for E1A and hexon gene expression. Late viral gene expression was present in some instances in the cells isolated from the lamnia propria derived from ileum and colon [10]. These data would, however, need further investigation to understand intestinal HAdV persistence and reactivation. The underlying mechanism in the intestine is so far unknown, but both immune cells and epithelial cells, capable of antigen presentation, are present in the gut.

## 4. HAdV Recombination—A Result of Intestinal HAdV Persistence?

There are several newly identified recombinant HAdV types isolated from stool specimens including mainly species D but also C types [48,57,58,59,60]. Whole genome sequencing of HAdV revealed a mixture of genes closely related to previously described types, e.g., the genes coding for the capsid proteins (hexon, fiber, and penton) and genes of the early transcribed proteins (E1, E3, and E4) combined in one new HAdV type. In addition, individual gene sequences were found to be novel and not closely related to any previous type. Moreover, detailed analysis of complete genome sequences of species B, C, and D types revealed a recombination of individual genome fragments within a species, and between species [59,61,62]. In most cases these recombinant types have been found in specimens from immunosuppressed patients like HSCT-recipients or AIDS patients [48,58]. It would be of great interest to find out whether the frequent persistence of the virus in the gut promotes recombination events resulting in the shedding of new HAdV types in stool samples of immunosuppressed patients. In immunocompetent individuals, persistent HAdV DNA has been found in about one-third of the investigated intestinal tissues derived from children and adults [2,10]. Additional screenings of intestinal biopsies, derived from the immunocompetent population with a larger number of patients, are still missing and would provide a more substantial overview of HAdV occurrence and persistence in the gut.

The finding of new recombinant species D types in the stool of adult immunosuppressed patients—including HAdV-D70, -D73, -D74, and -D75—with phylogenetic relationships between them supports the theory of intestinal recombination [48,63]. There are several computational analyses available discussing recombination events of human adenovirus sequences, mainly within HAdV species D [26,64] and homologous recombination requires co-infection of host cells with two or more types, which has been documented by data from analyzed stool samples [15,20]. A recent study showed similarity of HAdV species D recombination hot spots to bacterial Chi sequences. Moreover, upon in-vitro infection by HAdV-D19 and D29 in the presence of bacterial RecA enzyme, increased recombination between the viruses has been observed [65]. These valuable data indicate that adenovirus might implement bacterial recombination strategies and support the idea of intestinal adenoviral recombination events based on bacterial-viral interactions. It might be feasible that the intestine provides a site for recombination by a persistent co-infection or a combination of a persistent infection and an acute super-infection with another HAdV type of the same species. A possible trigger for the recombination could be the bacteria present in the gut and also the immunosuppression might play a role in HAdV recurrence from co-infections of persistent virus types.

## 5. Antiviral Therapy against HAdV Infections in the Gut

In immunosuppressed patients, especially in pediatric HSCT-recipients, the first detection of HAdV is mainly in stool specimens prior to other sites, and high virus loads above 10E6 viral DNA copies/g stool correlate with a significantly increased risk for viremia at a later time point [15,17,20,66]. Due to the European Conference on Infections in Leukaemia (ECIL) guidelines, therapy against HAdV infections in HSCT-recipients is recommended after first onset of viremia [67]. More recently, a preemptive antiviral treatment, already in the presence of HAdV positivity in stool with rapidly rising levels above a critical threshold, is under discussion [16]. Since early detection of the virus in stool specimens seems to reflect intestinal HAdV recurrence, antiviral approaches have to tackle the infection also in the gut. Unfortunately, available anti-adenoviral drugs used in HSCT-recipients, like cidofovir and ribavirin, show limited efficacy and toxicity. Cidofovir, a nucleotide analogue of deoxycytidine, inhibiting incorporation of deoxycytidine triphosphate into viral DNA leading to chain termination, is used as a preemptive anti-adenoviral therapy. However, due to poor cellular uptake and lower intracellular concentration, efficacy of cidofovir is compromised and the clearence of the virus is still dependent on T cell immune reconstitution [68,69]. Nevertheless, due to the absence of a more successful and approved antiviral therapy, cidofovir is the current standard treatment to control the viral replication and to prevent disseminated adenoviraemia. More recent approaches exploiting the cidofovir derivate brincidofovir or donor derived HAdV specific cytotoxic T cells (CTLs) have shown quite promising results [70], but there are some limitations. The cellular uptake of brincidofovir seems to be improved compared with cidofovir, but in several cases diarrhea occurred [71]. Moreover, since the survival of patients with HAdV viraemia is reportedly associated with recovery of the lymphocyte count and the presence of HAdV-specific T-cells [72], the use of immunotherapy with CTLs is shown to be successful in the reduction of HAdV infection in individual patients [73,74,75]. In addition, a very recent study showed that the capacity of adenovirus specific T cell response is dependent on the peptide epitope for stimulation which can be variable between different donors due to HLA type restriction [76]. This therapy is suggested for patients lacking virus-specific T cells and response to treatment with antivirals. However, the clinical applicability is limited by the access to enriched HAdV-specific T cells at a specific time point. In patients in whom the clearence of HAdV infection has been achieved, the immunotherapy is dependent on antigen presenting cells to stimulate T lymphocytes. HAdV specific T cells have been identified to consist mainly of CD4^+^ cell [4,77] and, interestingly, intestinal epithelial cells express MHC class II [78]. It is intriguing that immunosuppressed patients with frequently rising HAdV titers in the stool require therapy options acting against the virus in intestinal cells. The use of immortalized enterocytes or intestinal organoids might be excellent and suitable tools to screen potentially new antiviral compounds.

## 6. In-Vitro Model to Study Intestinal HAdV Infections

In-vitro infections with HAdV addressing viral entry, immune evasion, or any other deregulation of specific host cell signaling pathways are mainly performed in lung epithelial cell lines (A549) or in HEK293 cells [1]. The above mentioned prevalence of intestinal HAdV infections by different species and the frequent occurrence in the gut of immunocompromised patients, however, supports the notion that an intestinal cell model system might be of importance. Despite our knowledge of HAdV infections in the gastrointestinal tract, data about the use of colon cancer cell lines for in-vitro infections are rare [79] and conclusive studies are missing. A reduced expression of CAR in colon cancer cells compared with healthy colon tissue was reported [32], but further investigation might be necessary to understand the lack of in-vitro analyses with HAdV in colon cancer cells. There are limitations of conventional cell line cultures and a suitable in-vitro cell system to recapitulate tissue physiology in the setting of intestinal HAdV infections would have several benefits. Studying virus–host interactions or testing of new antivirals in the cell type where virus replication occurs in-vivo might be an advantage. HAdV-F40 and HAdV-F41 show very limited replication in A549 cells in comparison with the other HAdV types. A more recent approach exploiting enteroids provides a promising tool to study virus–host interactions in primary, intestinal epithelial cells. This three-dimensional tissue culture model has been shown to support the replication of several enteric viruses (e.g., rotaviruses, noroviruses, enteroviruses, and adenoviruses) [39,40,80,81,82,83]. The epithelial stem cells in enteroids can be cryopreserved and cultured for several months. Moreover, upon withdrawal of selective growth factors, they can be differentiated into various intestinal epithelial cells, including goblet cells, Paneth cells (derived from cells of the small intestine), and mature enterocytes [84,85]. The intestinal epithelial cells arrange themselves into a typical single cell layer by self-assembly and are permissive for HAdV infection and virus progeny production. Infection of enteroids derived from the small intestine showed efficient virus production for HAdV-C5, HAdV-B16, and HAdV-F41 [40] making them useful for any in-vitro infection studies with HAdV by providing an appropriate cell system.

## 7. Conclusions

Several insights about HAdV tropism by various species, potential mechanisms for virus persistence, and recombination between different types in the intestine are reviewed in this article. The summary of new ideas and approaches might be of interest for a better understanding of HAdV infections in the gut and to further improve the treatment of invasive infections under immunosuppression. However, there are still some unsolved questions for the future:

(I) Are immune modulating factors responsible for the species-specific replication capacity in the intestine? (II) Is HAdV reactivation possible in T lymphocytes of the lamnia propria? (III) Which cytokines play a role in supporting HAdV persistence in the gut? (IV) Do recombination events take place in the intestine upon immunosuppression? (V) Are there any characteristics of HAdV infected intestinal cells compared with other epithelial cells, also in regard to therapy success?

## Figures and Tables

**Table 1:** Current taxonomy of human adenoviruses.

Species	Types ^a^
A	12, 18, 31, *61*
B	3, 7, 11, 14, 16, 21, 34, 35, 50, 55, *66, 68, 76–79*
C	1, 2, 5, 6, *57, 89*
D	8–10, 13, 15, 17, 19, 20, 22–30, 32, 33, 36–39, 42–49, 51, *53, 54, 56, 58–60, 63–65, 67, 69–75, 80–88, 90–103*
E	4
F	40, 41
G	*52*

^a^ The types indicated in italics were identified by genomic and not by serological analyses.
